# Child maltreatment and eating disorders among men and women in adulthood: Results from a nationally representative United States sample

**DOI:** 10.1002/eat.22783

**Published:** 2017-10-09

**Authors:** Tracie O. Afifi, Jitender Sareen, Janique Fortier, Tamara Taillieu, Sarah Turner, Kristene Cheung, Christine A. Henriksen

**Affiliations:** ^1^ Department of Psychiatry University of Manitoba Manitoba R3E 0W5 Canada; ^2^ Department of Community Health Sciences University of Manitoba Manitoba R3E 0W5 Canada; ^3^ Applied Health Sciences Program, University of Manitoba Manitoba R3E 0W5 Canada; ^4^ Department of Psychology University of Manitoba Manitoba R3E 0W5 Canada; ^5^ Department of Clinical Health Psychology University of Manitoba Manitoba R3E 0W5 Canada

**Keywords:** bulimia nervosa, binge‐eating disorder, anorexia nervosa, child abuse, child, maltreatment, neglect

## Abstract

**Objective:**

Child maltreatment is associated with an increased likelihood of having mood disorders, anxiety disorders, post‐traumatic stress disorder, substance use disorders, and personality disorders, but far less is known about eating disorders. The objective of the current study was to examine the associations between child maltreatment, including harsh physical punishment, physical abuse, sexual abuse, emotional abuse, emotional neglect, physical neglect, and exposure to intimate partner violence, and eating disorders in adulthood among men and women.

**Method:**

Data were from the National Epidemiologic Survey on Alcohol and Related Conditions wave 3 (NESARC‐III) collected in 2012–2013. The sample was nationally representative of the United States adult population (*N* = 36,309). Lifetime eating disorders (anorexia nervosa [AN], bulimia nervosa [BN], and binge‐eating disorder [BED]) were assessed using diagnostic and statistical manual of mental disorders, fifth edition (DSM‐5) criteria and the alcohol use disorder and associated disabilities interview schedule‐5 (AUDADIS‐5).

**Results:**

The prevalence of any lifetime eating disorder was 1.7% (0.8% among men and 2.7% among women). All child maltreatment types were associated with AN, BN, and BED with notable differences among men and women. Overall, the types of child maltreatment with the strongest relationships with any eating disorder were sexual abuse and physical neglect among men and sexual abuse and emotional abuse among women.

**Discussion:**

Clinicians should be mindful that child maltreatment experiences are associated with increased odds of eating disorders including AN, BED, and BN. Such relationships are significant among men and women although notable gender differences in these relationships exist. Abstract word count = 248.

## 1 INTRODUCTION

Child maltreatment has been identified as an important public health problem worldwide (Gilbert et al., 2009). Child maltreatment includes harsh physical punishment, physical abuse, sexual abuse, emotional abuse, emotional neglect, physical neglect, and exposure to intimate partner violence (IPV). It is well established that child maltreatment is associated with mood disorders, anxiety disorders, post‐traumatic stress disorder (PTSD), substance use disorders, and personality disorders across the lifespan (Afifi et al., 2008, 2014, 2011; Afifi, Brownridge, Cox, & Sareen, 2006; Afifi, Henriksen, Asmundson, & Sareen, 2012; Afifi, Mota, Dasiewicz, MacMillan, & Sareen, 2012; Kessler, Davis, & Kendler, 1997; Kessler et al., 2010; MacMillan et al., 1999, 2001; McLaughlin et al., 2010; Scott, Smith, & Ellis, 2010). Although the literature on child maltreatment and mental disorders is relatively large, the number of studies published on child maltreatment and eating disorders is comparatively small.

We know from clinical samples that a high proportion of patients with eating disorders also report experiencing child maltreatment (Smolak & Murnen, 2002). For example, among women with binge‐eating disorder (BED), 52% reported emotional abuse, 28% reported physical abuse, 31% reported sexual abuse, 68% reported emotional neglect, and 48% reported physical neglect (Becker & Grilo, 2011). However, these clinical data are limited because they likely only include the most severe cases of eating disorders and experiences of child maltreatment. As well, selection bias results when using clinical cases known as Berkson's bias, which indicates that bias is inherent when assessing relationships in clinical samples, with exposure and disease increasing the likelihood of treatment (Berkson, 1946).

There is a dearth of epidemiologic studies examining the association between child maltreatment and eating disorders using representative community samples of men and women; of these, some studies are dated (Garfinkel, Kennedy, & Kaplan, 1995), have included eating behaviors and not eating disorder diagnoses (Sachs‐Ericsson et al., 2012), only examined one eating disorder (Garfinkel et al., 1995), only examined sexual abuse (Micali et al., 2017), or only included women (Micali et al., 2017). Few eating disorder studies have been conducted among men using representative general population data (Gadalla, 2009; Woodside et al., 2001), making more research necessary (Cottrell & Williams, 2016; Woodside et al., 2004).

What we know from these few population‐based studies is that sexual abuse and physical abuse are related to an increased likelihood of experiencing eating disorders or symptoms (Garfinkel et al., 1995; Micali et al., 2017; Sachs‐Ericsson et al., 2012). More studies using representative community samples are necessary since clinical data alone are not adequate for understanding the scope of the problem or to inform prevention strategies. Furthermore, literature reviews have indicated that sexual abuse and physical abuse are related to eating disorders, but less is known about emotional abuse (Caslini et al., 2015) and emotional and physical neglect (Pignatelli, Wampers, Loriedo, Biondi, & Vanderlinden, 2017), and no studies have adequately examined how associations with child maltreatment may vary for different eating disorders. In addition, when examining the relationship between child maltreatment and eating disorders, it is necessary to consider possible confounding factors. Previous research has indicated that eating disorders are highly comorbid with other mental disorders (Friborg et al., 2014; Grucza, Przybeck, & Cloninger, 2007; Hudson et al., 2007; Martinussen et al., 2017) and family history of dysfunction such as parental psychopathology or substance use (Sachs‐Ericsson et al., 2012). As well, some studies have found some significant differences in eating disorders according to age, education, and marital status (Hudson et al., 2007; Kessler et al., 2013). In contrast, a recent study indicated that eating disorder symptoms did not vary according to income, education, and ethnicity (Mulders‐Jones, Mitchison, Girosi, & Hay, 2017). It is important to adjust for these factors in future studies because they are related to child maltreatment and potentially related to eating disorders.

Overall, the child maltreatment and eating disorder literature is often limited by the use of unrepresentative samples (i.e., clinical samples, college samples, convenience community samples) that restricts generalizability of the findings (Becker & Grilo, 2011; Burns, Fischer, Jackson, & Harding, 2012; Fosse & Holen, 2006; Grilo & Masheb, 2001; Groleau et al., 2012; Guillaume et al., 2016; Hund & Espelage, 2006; Kennedy, Ip, Samra, & Gorzalka, 2007; Kong & Bernstein, 2009; Moulton, Newman, Power, Swanson, & Day, 2015), the inclusion of only one type of child maltreatment (Chen et al., 2010; Groleau et al., 2012; Hund & Espelage, 2006; Micali et al., 2017) or one type of eating disorder (Becker & Grilo, 2011; Burns, Fischer, Jackson, & Harding, 2012; Carter, Bewell, Blackmore, & Woodside, 2006; Groleau et al., 2012), the lack of adjustment for potential confounding factors such as comorbid mental disorders or family history of dysfunction, and the inclusion of only women or girls in research (Becker & Grilo, 2011; Cachelin, Schug, Juarez, & Monreal, 2005; Fosse & Holen, 2006; Mazzeo & Espelage, 2002; Micali et al., 2017; Rayworth, Wise, & Harlow, 2004; Romans, Gendall, Martin, & Mullen, 2001; Sanci et al., 2008; Striegel‐Moore, Dohm, Pike, Wilfley, & Fairburn, 2002). As such, very little is known about how child maltreatment types are associated with each eating disorder among men and women in the general population and if relationships vary according to gender. It is also not currently known if child maltreatment types are more strongly associated with certain types of eating disorders.

The specific research objectives of the current study were to: (a) estimate the lifetime diagnostic and statistical manual of mental disorders, fifth edition (DSM‐5) prevalence of eating disorders; (b) confirm if lifetime eating disorders are more likely among women compared to men in the general population using new DSM‐5 criteria; (c) examine the relationships between sociodemographic variables, comorbid mental disorders, a family history of dysfunction, and child maltreatment with anorexia nervosa (AN), bulimia nervosa (BN), BED, and any eating disorder among the whole sample (men and women combined); (d) examine the associations between child maltreatment types and eating disorders in adulthood separately among men and women and after adjusting for sociodemographic variables, a family history of dysfunction, and mental disorders; and (e) determine if child maltreatment types are more strongly associated with certain eating disorders.

## 2 METHOD

### 2.1 Data and sample

Data for the study were from the National Epidemiologic Survey on Alcohol and Related Conditions Wave 3 (NESARC‐III) collected in 2012–2013 (*N* = 36,309) using in‐person interviews. The NESARC‐III used a multistage probability sample design to randomly select a representative sample of civilian, noninstitutionalized adults in the US (aged 18 and older; response rate = 60.1%; mean age = 46.5 years). In the sample, the mean age of men and women was 45.9 years (*SD* = 0.2) and 47.2 years (*SD* = 0.2), respectively. Previous publications are available for more information regarding the NESARC‐III (Grant, Goldstein, Saha, et al., 2015; Grant, Goldstein, Smith, et al., 2015) and the original NESARC samples (Grant et al., 2003; Hasin & Grant, 2015; Ruan et al., 2008). The Westat Institutional Review Board and the Combined Neuroscience Institutional Review Board of the National Institutes of Health approved the data collection.

### 2.2 Measurement

The alcohol use disorder and associated disabilities interview schedule‐5 (AUDADIS‐5) was used to assess lifetime DSM‐5 (American Psychiatric Association, 2013) eating disorders, which included AN, BED, and BN. The AUDADIS‐5 is a fully structured interview and was designed to measure DSM‐5 criteria alcohol use disorders, nicotine use disorders, drug use disorders, and several mood, anxiety, trauma‐related, eating, and personality disorders (Grant, Goldstein, Saha, et al., 2015). The reliability and validity of past‐year substance use, mood, anxiety, and trauma, and stress‐related disorders using this tool have been published (Grant, Goldstein, Saha, et al., 2015) but past‐year eating disorder diagnoses, specifically, could not be evaluated because of the low prevalence of eating disorders. However, the test–retest design has indicated that the inter‐rater reliability of DSM‐5 diagnoses and dimensional scales in the NESARC‐III using the AUDADIS‐5 for assessing substance use were good to excellent (Kappa ranged from 0.40 to 0.87 and intraclass correlation ranging from 0.45 to 0.85) and fair to good for mood, anxiety, personality, trauma and stress‐related disorders (Kappa ranged from 0.35 to 0.54 and intraclass correlation ranging from 0.50 to 0.79) (Grant, Goldstein, Smith, et al., 2015). With regard to procedural validity through clinician re‐appraisal, substance use disorders were good (Kappa range = 0.40–0.72) with excellent concordance on dimensional scales (ICC ≥ 0.75) (Hasin, Greenstein, et al., 2015). Mood disorders, anxiety disorders, and PTSD had moderate validity (Kappa range = 0.24–0.59) with good concordance on dimensional scales (ICC ≥ 0.53–0.81) (Hasin, Shmulewitz, et al., 2015). It has been concluded that the AUDADIS‐5 is a useful tool for studying mental disorders using survey methods of the general population (Grant, Goldstein, Smith, et al., 2015; Hasin, Greenstein, et al., 2015; Hasin, Shmulewitz, et al., 2015).

The modules designed to assess DSM‐5 criteria for eating disorders contained 10–16 questions for each eating disorder. For AN, a number of questions were asked about height and weight to determine less than minimally normal weight followed by questions asking about restricting food intake, behaviors to control weight, being afraid to gain weight, and perceptions of one's body (i.e., think you look fat, weight, or body shape is very important, do you think your weight might have been unhealthy). For BN, a binge‐eating episode was assessed by asking two questions as follows: “Have you ever eaten an usually large amount (more than most people would eat) of food within any 2‐hr period, not including the holidays?” and “Was there ever a time that you ate an unusually large amount of food at least once a week for at least 3 months?” Additionally, several questions were asked about loss of control (e.g., did you feel that you couldn't stop eating or control how much you were eating, find that you ate until you felt uncomfortably full), two questions asked about methods to control weight (e.g., vomiting, using enemas, laxatives, diuretics, other medicines, fasting, or excessive exercise) and occurrence of these methods, and questions were asked regarding self‐evaluation. For BED, binge‐eating episodes and loss of control were measured using the same questions as BN. In addition, several questions were asked about distress (e.g., feel disgusted with yourself, depressed or very guilty about eating so much) and occurrence of binge‐eating episodes (at least once a week for 3 months). Algorithms based on DSM‐5 criteria were used to compute the presence or absence of lifetime AN, BN, BED, and any eating disorders.

Seven types of child maltreatment (harsh physical punishment, physical abuse, sexual abuse, emotional abuse, emotional neglect, physical neglect, and exposure to IPV) before the age of 18 years were assessed in the NESARC‐III using questions adapted from the adverse childhood experiences study (Dong, Anda, Dube, Giles, & Felitti, 2003; Dube et al., 2003), which included a subset of questions from the conflict tactics scales (Straus, 1979; Straus, Hamby, Boney‐McCoy, & Sugarman, 1996) and the childhood trauma questionnaire (Bernstein et al., 1994). Using a subset of questions is a commonly used method to assess child maltreatment in large epidemiologic surveys. All questions assessing harsh physical punishment, physical abuse, sexual abuse, emotional abuse, physical neglect, and exposure to IPV were based on a 5‐point ordinal scale (*never*, *almost never*, *sometimes*, *fairly often*, and *very often*). Consistent with previous publications (Afifi, Mota, MacMillan, & Sareen, 2013; Afifi, Mota, et al., 2012), respondents who reported an answer of *sometimes* or greater on the question, “Before you were 18, how often did a parent/other adult living in your home push, grab, shove, slap or hit you” were categorized as having experienced harsh physical punishment. Physical abuse was defined as being hit so hard that it left bruises or caused an injury (i.e., any response other than *never*). Sexual abuse was defined as having ever experienced any unwanted sexual touching or fondling and/or attempted or completed sexual intercourse by an adult or other person (i.e., any response other than *never*). Emotional abuse was defined as having a parent or other adult living in the home swear at, insult, say hurtful things, threaten to hit, or throw something at the respondent, or act in any other way that made the respondent afraid of being physically hurt (any occurring *fairly often* or *very often*). Emotional neglect was assessed with five questions inquiring about how often the respondent felt part of a close‐knit family, that the respondent's family was a source of strength and support, that a family member wanted the respondent to succeed, made the respondent feel important or special, or believed in the respondent. Emotional neglect questions were assessed with ordinal response categories of *never true, rarely true, sometimes true, often true, and very often true*. According to the coding guidelines of the tool, and as used in previous studies (Afifi et al., 2011; Dong et al., 2003; Dube et al., 2003), the questions were then reverse‐coded and summed. A dichotomous variable was then computed with scores of 15 or greater categorized as having experienced emotional neglect. Physical neglect was defined as having ever been left alone or unsupervised before the age of 10 years or going without necessary clothing, school supplies, food, or medical treatment (i.e., any response other than *never*). Exposure to IPV was defined as the respondent's mother or other female adult in the home ever being physically abused, pushed, grabbed, slapped, kicked, bit, hit with a fist or something hard, repeatedly hit for at least a few minutes, threatened with a knife or gun, or hurt with a knife or gun by the respondent's father or other adult male (i.e., any response other than *never*). A dichotomous variable was computed to determine the presence or absence of any of the previously described types of child maltreatment. As well, a three‐level ordinal count variable was computed to determine the number of child maltreatment types experienced by the respondents (0, 1, and 2 or more). This was the largest number of categories that could be computed for the count variable because of low cell sizes for increasing numbers of child maltreatment types experienced, particularly among those with BN.

#### 2.2.1 Covariates

Sociodemographic covariates included age, past‐year household income, marital status, visible minority (White vs.Black/American Indian/Alaska Native/Asian/Native Hawaiian/Other Pacific Islander, Hispanic), and education. Family history of dysfunction (Dong et al., 2003; Dube et al., 2003) included a parent or other adult living in their home experiencing before age 18 years: (a) problems with alcohol, (b) problems with drugs, (c) incarceration, (d) being treated or hospitalized for a mental illness, (e) attempted suicide, and (f) death by suicide. Any lifetime mental disorder variable was computed based on whether the respondent met DSM‐5 (American Psychiatric Association, 2013) criteria for any of the following: major depression, dysthymia, manic episode, hypomanic episode, panic disorder, agoraphobia, social phobia, specific phobia, generalized anxiety disorder, PTSD, conduct disorder, schizotypal personality disorder, borderline personality disorder, antisocial personality disorder, and substance use disorders (alcohol use disorder, nicotine use disorder, and drug use disorder).

### 2.3 Statistical analysis

Statistical weights were computed for the dataset and applied in all models to ensure the NESARC‐III data corrected for oversampling and were representative of the US general population. Taylor series linearization was used as a variance estimation technique to account for the complex sampling design. First, descriptive and univariate statistics were computed. Second, multivariable logistic regressions were computed to understand the relationships between child maltreatment types and eating disorders while adjusting for important covariates. More specifically, a series of nested multivariable logistic regression models were computed for men and women for child maltreatment types and eating disorders, first adjusting for sociodemographic variables and family history of dysfunction (Adjusted Odds Ratio [AOR‐1]), second adjusting for all variables in AOR‐1 and any mental disorder (AOR‐2), and third adjusting for all variables in AOR‐2 and each child maltreatment type (AOR‐3) entered into one model. Because of low prevalence of BN, logistic regression models could only be run for AOR‐1 among men. All adjusted models could be computed among women. Gender and child maltreatment interactions terms were also computed to determine if gender modified the relationships between child maltreatment and eating disorder types. Finally, multinomial logistic regressions were computed adjusting for gender to determine if child maltreatment types were more strongly associated with certain eating disorders. In the multinomial models, the odds ratios indicate the odds of having AN, BN, or BED compared to no eating disorder related to each child maltreatment type. We also compared the odds between AN, BN, and BED (significant differences represented using superscripts) in the models to determine if there were significant differences among each eating disorder.

## 3 RESULTS

The prevalence of any lifetime eating disorder in the whole sample was 1.7% (*n* = 617) (0.8% [*n* = 128] among men and 2.7% [*n* = 489] among women). The prevalence of each individual lifetime eating disorder was 0.8% (*n* = 276) for AN (0.2% among men [*n* = 36] and 1.4% [*n* = 240] among women), 0.2% for BN (*n* = 82) (0.07% among men [*n* = 11] and 0.4% [*n* = 71] among women), and 0.8% (*n* = 285) for BED (0.5% [*n* = 82] among men and 1.0% [*n* = 203] among women). Women compared to men were more likely to experience AN, BN, BED, and any eating disorder (see Table 1). No significant age differences were noted except for any lifetime eating disorder, AN, and BN being less likely among individuals aged 50 years and older compared to those aged 18 to 29 years. Highest education categories were associated with increased odds of having any eating disorder and AN compared to respondents who had not completed high school. Being single or never‐married compared to being married was associated with increased odds of BN. Differences in household income were not found across eating disorders types. Family history of dysfunction and mental disorders were associated with increased odds of all eating disorders. As well, all child maltreatment types were associated with increased odds of all eating disorders.

**Table 1:** Study cohort characteristics

	Anorexia nervosa		Bulimia nervosa		Binge‐eating disorder		Any eating disorder	
Covariate	% (*n*)	OR (95% CI)	% (*n*)	OR (95% CI)	% (*n*)	OR (95% CI)	% (*n*)	OR (95% CI)
Sex								
Men	11.4 (36)	1.00	13.8 (11)	1.00	31.3 (82)	1.00	20.7 (128)	1.00
Women	88.6 (240)	7.28*** (4.64,11.42)	86.2 (71)	5.81*** (2.66, 12.67)	68.7 (203)	2.05*** (1.51, 2.78)	79.3 (489)	3.62*** (2.83, 4.63)
Age, years								
18–29	28.0 (76)	1.00	30.1 (23)	1.00	20.8 (65)	1.00	24.8 (155)	1.00
30–39	19.9 (61)	0.92 (0.65, 1.31)	27.9 (25)	1.20 (0.61, 2.38)	17.1 (54)	1.06 (0.66, 1.71)	19.3 (132)	1.01 (0.76, 1.33)
40–49	22.8 (55)	0.98 (0.67, 1.43)	25.7 (21)	1.02 (0.47, 2.24)	19.3 (52)	1.11 (0.71, 1.74)	21.9 (124)	1.06 (0.79, 1.44)
50+	29.2 (84)	0.52*** (0.36, 0.74)	16.3 (13)	0.27** (0.10, 0.72)	42.8 (114)	1.03 (0.70, 1.51)	34.1 (206)	0.68* (0.50, 0.92)
Marital status								
Married/common law	58.3 (138)	1.00	44.1 (33)	1.00	55.2 (124)	1.00	55.6 (283)	1.00
Widowed/divorced/separated	19.4 (72)	0.98 (0.69, 1.39)	22.3 (20)	1.49 (0.77, 2.89)	19.6 (72)	1.04 (0.76, 1.43)	19.7 (156)	1.05 (0.82, 1.33)
Single/never married	22.3 (66)	0.98 (0.67, 1.42)	33.6 (29)	1.96* (1.03, 3.76)	25.3 (89)	1.18 (0.85, 1.64)	24.7 (178)	1.14 (0.88, 1.49)
Race/ethnicity								
White	80.0 (210)	1.00	70.8 (45)	1.00	70.9 (179)	1.00	74.4 (415)	1.00
Other	20.0 (66)	0.49*** (0.34, 0.69)	29.2 (37)	0.81 (0.43, 1.52)	29.1 (106)	0.80 (0.58, 1.10)	25.6 (202)	0.67*** (0.54, 0.83)
Past year household income, $								
≤19,999	18.2 (64)	1.00	20.7 (23)	1.00	23.1 (86)	1.00	20.8 (167)	1.00
20,000–39,999	16.1 (54)	0.76 (0.48, 1.22)	17.8 (23)	0.74 (0.40, 1.37)	24.8 (78)	0.93 (0.67, 1.28)	20.0 (150)	0.83 (0.65, 1.06)
40,000–69,999	22.7 (58)	1.13 (0.77, 1.65)	24.2 (15)	1.06 (0.46, 2.41)	20.8 (61)	0.81 (0.58, 1.13)	21.5 (127)	0.93 (0.73, 1.19)
>70,000	43.0 (100)	1.49 (0.98, 2.28)	37.3 (21)	1.14 (0.53, 2.45)	31.3 (60)	0.85 (0.59, 1.23)	37.7 (173)	1.14 (0.88, 1.48)
Education								
Less than high school	4.7 (14)	1.00	11.7 (14)	1.00	12.8 (40)	1.00	9.1 (66)	1.00
High school	16.5 (48)	1.78 (0.88, 3.60)	19.2 (16)	0.82 (0.31, 2.16)	24.5 (67)	0.96 (0.61, 1.54)	20.3 (126)	1.12 (0.78, 1.63)
Some college	28.6 (74)	3.70*** (1.81, 7.56)	23.4 (18)	1.20 (0.55, 2.64)	22.8 (65)	1.07 (0.69, 1.68)	25.1 (150)	1.67** (1.20, 2.34)
Completed postsecondary degree	50.2 (140)	3.54*** (1.92, 6.51)	45.7 (34)	1.28 (0.57, 2.89)	39.9 (113)	1.02 (0.68, 1.53)	45.5 (275)	1.66*** (1.23, 2.23)
Any family history of dysfunction								
No	54.1 (149)	1.00	43.7 (36)	1.00	43.5 (125)	1.00	49.2 (300)	1.00
Yes	45.9 (122)	2.05*** (1.56, 2.68)	56.3 (44)	3.10*** (1.83, 5.23)	56.5 (157)	3.15*** (2.28, 4.34)	50.8 (308)	2.53*** (2.11, 3.03)
Any mental disorders								
No	14.4 (39)	1.00	6.3 (7)	1.00	5.4 (18)	1.00	9.5 (60)	1.00
Yes	85.6 (237)	4.29*** (2.90, 6.34)	93.7 (75)	10.59*** (4.56, 24.63)	94.6 (267)	12.73*** (7.22, 22.42)	90.5 (557)	6.95*** (5.07, 9.53)
Harsh physical punishment								
No	72.4 (192)	1.00	66.8 (50)	1.00	64.5 (180)	1.00	68.9 (408)	1.00
Yes	27.6 (83)	1.73*** (1.29, 2.34)	33.2 (32)	2.26** (1.25, 4.10)	35.5 (105)	2.52*** (1.88, 3.36)	31.1 (208)	2.08*** (1.71, 2.53)
Physical abuse								
No	69.5 (188)	1.00	69.5 (52)	1.00	62.7 (177)	1.00	67.5 (404)	1.00
Yes	30.5 (87)	2.07*** (1.50, 2.87)	30.5 (30)	2.06** (1.21, 3.52)	37.3 (108)	2.82*** (2.14, 3.70)	32.5 (212)	2.29*** (1.87, 2.81)
Sexual abuse								
No	70.7 (191)	1.00	66.1 (46)	1.00	69.0 (193)	1.00	70.6 (421)	1.00
Yes	29.3 (78)	3.31*** (2.41, 4.54)	33.9 (32)	4.06*** (2.39, 6.89)	31.0 (89)	3.59*** (2.60, 4.96)	29.4 (184)	3.38*** (2.72, 4.19)
Emotional abuse								
No	75.5 (204)	1.00	70.2 (50)	1.00	68.8 (191)	1.00	73.0 (432)	1.00
Yes	24.5 (71)	2.86*** (2.01, 4.06)	29.8 (32)	3.72*** (2.16, 6.42)	31.2 (93)	4.02*** (2.91, 5.56)	27.0 (183)	3.32*** (2.65, 4.17)
Emotional neglect								
No	86.1 (228)	1.00	72.0 (52)	1.00	81.6 (231)	1.00	82.8 (493)	1.00
Yes	13.9 (46)	1.46* (1.03, 2.06)	28.0 (29)	3.53*** (2.00, 6.24)	18.4 (51)	2.05*** (1.49, 2.81)	17.2 (118)	1.90*** (1.52, 2.38)
Physical neglect								
No	62.6 (168)	1.00	57.4 (43)	1.00	48.7 (134)	1.00	56.5 (334)	1.00
Yes	37.4 (107)	1.56*** (1.22, 2.00)	42.6 (39)	1.93* (1.11, 3.35)	51.3 (151)	2.76*** (2.11, 3.62)	43.5 (282)	2.03*** (1.68, 2.46)
Exposure to intimate partner violence								
No	68.5 (186)	1.00	63.5 (51)	1.00	60.9 (164)	1.00	65.5 (389)	1.00
Yes	31.5 (86)	2.11*** (1.51, 2.96)	36.5 (31)	2.63** (1.44, 4.81)	39.1 (116)	2.96*** (2.23, 3.92)	34.5 (219)	2.45*** (2.02, 2.97)
Any child maltreatment								
No	36.0 (97)	1.00	31.7 (21)	1.00	26.4 (68)	1.00	31.8 (181)	1.00
Yes	64.0 (177)	1.86*** (1.39, 2.50)	68.3 (61)	2.25** (1.25, 4.06)	73.6 (217)	2.92*** (2.16, 3.94)	68.2 (434)	2.27*** (1.85, 2.78)
Number of types of child maltreatment								
0	36.2 (97)	1.00	32.1 (21)	1.00	26.7 (68)	1.00	32.1 (181)	1.00
1	17.4 (45)	1.15 (0.76, 1.73)	15.4 (10)	1.14 (0.40, 3.27)	20.0 (54)	1.79** (1.18, 2.70)	18.6 (106)	1.39* (1.04, 1.85)
2+	46.4 (130)	2.46*** (1.79, 3.36)	52.6 (50)	3.12*** (1.72, 5.67)	53.2 (158)	3.82*** (2.78, 5.25)	49.3 (320)	2.98*** (2.40, 3.70)

*Note*. Data are presented as % (*n*). All *n* values were unweighted, and all percentages were weighted.

*p ≤ .05; **p ≤ .01; ***p ≤ .001.

The results of analyses examining the associations between child maltreatment and any lifetime eating disorder are presented in Table 2. Among men, harsh physical punishment, physical abuse, sexual abuse, emotional abuse, physical neglect, exposure to IPV, and any child maltreatment were associated with increased odds of any eating disorder after adjusting for sociodemographic variables and family history of dysfunction (AOR‐1 ranged from 1.56 to 2.92). When further adjusting for any mental disorder, odds ratios remained significantly associated with any eating disorder among men for sexual abuse, emotional abuse, physical neglect, exposure to IPV, and any child maltreatment (AOR‐2 ranged from 1.66 to 2.43). When also adjusting for the co‐occurrence of child maltreatment types, sexual abuse (AOR‐3 = 2.14; 95% CI 1.27–3.60) and physical neglect (AOR‐3 = 1.58; 95% CI 1.05–2.35) remained independently associated with any eating disorder among men.

**Table 2:** Associations between types of child maltreatment and any lifetime eating disorder in men and women

	Men					Women				
Child Maltreatment type	Lifetime prevalence (no eating disorder) % (*n*)	Lifetme prevalence (eating disorder) % (*n*)	AOR‐1	AOR‐2	AOR‐3	Lifetime prevalence (no eating disorder) % (*n*)	Lifetme prevalence (eating disorder) % (*n*)	AOR‐1	AOR‐2	AOR‐3
Harsh physical punishment	18.4 (2953)	30.6 (45)	1.56* (1.08, 2.27)	1.28 (0.87, 1.87)	0.87 (0.58, 1.31)	17.3 (3515)	31.2 (163)	1.83*** (1.45, 2.31)	1.51*** (1.19, 1.91)	0.83 (0.61, 1.14)
Physical abuse	17.9 (2881)	32.6 (45)	1.75** (1.15, 2.67)	1.40 (0.92, 2.13)	0.89 (0.53, 1.50)	16.8 (3409)	32.4 (167)	2.01*** (1.59, 2.55)	1.69*** (1.34, 2.14)	1.26 (0.91, 1.75)
Sexual abuse	6.0 (970)	18.6 (23)	2.92*** (1.67, 5.11)	2.43** (1.40, 4.24)	2.14** (1.27, 3.60)	15.7 (3143)	32.2 (161)	2.27*** (1.75, 2.94)	1.85*** (1.43, 2.41)	1.58** (1.19, 2.11)
Emotional abuse	8.8 (1451)	21.5 (32)	2.03** (1.26, 3.26)	1.66* (1.04, 2.64)	1.23 (0.70, 2.16)	11.2 (2245)	28.4 (151)	2.48*** (1.88, 3.26)	2.02*** (1.52, 2.67)	1.66* (1.12, 2.45)
Emotional neglect	8.7 (1474)	14.3 (17)	1.46 (0.79, 2.70)	1.38 (0.74, 2.55)	1.07 (0.52, 2.18)	10.9 (2314)	18.0 (101)	1.63*** (1.23, 2.15)	1.42* (1.08, 1.88)	0.93 (0.67, 1.30)
Physical neglect	30.0 (4794)	52.1 (68)	2.19*** (1.49, 3.23)	1.87** (1.27, 2.76)	1.58* (1.05, 2.35)	25.2 (5110)	41.3 (214)	1.74*** (1.39, 2.17)	1.50*** (1.20, 1.87)	1.15 (0.86, 1.53)
Exposure to intimate partner violence	16.3 (2630)	35.1 (48)	2.00** (1.28, 3.13)	1.75* (1.12, 2.73)	1.51 (0.91, 2.49)	19.0 (3875)	34.4 (171)	1.75*** (1.36, 2.25)	1.53*** (1.20, 1.95)	1.14 (0.85, 1.53)
Any child maltreatment	49.3 (7829)	70.6 (93)	2.09** (1.31, 3.33)	1.66* (1.03, 2.66)	—	48.0 (9599)	67.6 (341)	1.93*** (1.50, 2.48)	1.57*** (1.21, 2.02)	—
Number of types of child maltreatment	—	—	—	—	—	—	—	—	—	—
0	51.1 (7710)	29.4 (35)	1.00	1.00	—	52.3 (10047)	32.8 (146)	1.00	1.00	—
1	23.1 (3555)	27.1 (32)	1.94* (1.12, 3.35)	1.65 (0.95, 2.87)	—	20.2 (3965)	16.3 (74)	1.20 (0.85, 1.69)	1.04 (0.74, 1.47)	—
2+	25.8 (4175)	43.5 (61)	2.28** (1.36, 3.82)	1.69* (1.01, 2.85)	—	27.5 (5505)	50.9 (259)	2.52*** (1.92, 3.32)	1.94*** (1.47, 2.57)	—

*Note*. All *n* values were unweighted, and all percentages were weighted. AOR‐1, adjusted for household income, age (continuous), marital status, race/ethnicity, education, and family history of dysfunction. AOR‐2, adjusted for household income, age (continuous), marital status, race/ethnicity, education, family history of dysfunction, and any mental disorder. AOR‐3, adjusted for household income, age (continuous), marital status, race/ethnicity, education, family history of dysfunction, any mental disorder, and all other types of child maltreatment.

*p ≤ .05; **p ≤ .01; ***p ≤ .001.

Among women, all individual child maltreatment types and any child maltreatment were associated with increased odds of any lifetime eating disorder in AOR‐1 models (AOR‐1 ranged from 1.63 to 2.48). When further adjusting for any mental disorder, all odds ratios remained statistically significant (AOR‐2 ranged from 1.42 to 2.02). When also adjusting for the co‐occurrence of child maltreatment types, sexual abuse (AOR‐3 = 1.58; 95% CI 1.19–2.11) and emotional abuse (AOR‐3 = 1.66; 95% CI 1.12–2.45; *p* = .011) remained significantly associated with any eating disorder among women. A general trend was found for men and women with increasing number of child maltreatment types corresponding with significantly increased odds of having an eating disorder (although not all models were statistically significant).

Table 3 provides the findings for AN. Among men, physical abuse, sexual abuse, physical neglect, and exposure to IPV were associated with increased odds of AN in AOR‐1 models (AOR‐1 ranged from 2.39 to 4.51). In AOR‐2, odds ratios were attenuated, but remained significant for physical abuse, sexual abuse, and exposure to IPV (AOR‐2 ranged from 2.93 to 3.78). When further adjusting for the co‐occurrence of other types of child maltreatment, sexual abuse (AOR‐3 = 2.90; 95% CI 1.26–6.70) remained independently associated with AN among men.

**Table 3:** Associations between types of child maltreatment and lifetime anorexia nervosa in men and women

	Men					Women				
Child maltreatment type	Lifetime prevalence no anorexia nervosa % (*n*)	Lifetime prevalence anorexia nervosa % (*n*)	AOR‐1	AOR‐2	AOR‐3	Lifetime prevalence no anorexia nervosa % (*n*)	Lifetime prevalence anorexia nervosa % (*n*)	AOR‐1	AOR‐2	AOR‐3
Harsh physical punishment	18.5 (2984)	32.8 (14)	1.94 (0.99, 3.81)	1.63 (0.82, 3.27)	0.68 (0.29, 1.57)	17.6 (3609)	26.9 (69)	1.54* (1.10, 2.14)	1.32 (0.94, 1.85)	0.84 (0.52, 1.34)
Physical abuse	18.0 (2910)	45.2 (16)	3.55*** (1.69, 7.48)	2.93** (1.36, 6.29)	2.03 (0.85, 4.88)	17.0 (3505)	28.6 (71)	1.73** (1.19, 2.52)	1.52* (1.04, 2.21)	1.24 (0.70, 2.18)
Sexual abuse	6.0 (984)	24.6 (9)	4.51** (1.71, 11.88)	3.78** (1.44, 9.91)	2.90* (1.26, 6.70)	15.9 (3235)	29.9 (69)	2.20*** (1.48, 3.29)	1.89** (1.27, 2.80)	1.77* (1.13, 2.79)
Emotional abuse	8.8 (1474)	21.8 (9)	2.21 (0.96, 5.10)	1.85 (0.79, 4.31)	0.97 (0.31, 3.01)	11.5 (2334)	24.8 (62)	2.08*** (1.41, 3.08)	1.77** (1.19, 2.64)	1.60 (0.86, 2.97)
Emotional neglect	8.8 (1486)	16.3 (5)	2.07 (0.64, 6.72)	2.05 (0.63, 6.65)	1.34 (0.39, 4.60)	11.1 (2374)	13.6 (41)	1.21 (0.79, 1.85)	1.08 (0.71, 1.64)	0.75 (0.45, 1.24)
Physical neglect	30.1 (4844)	54.0 (18)	2.39* (1.09, 5.24)	2.08 (0.94, 4.58)	1.31 (0.62, 2.75)	25.5 (5235)	35.3 (89)	1.35* (1.04, 1.77)	1.20 (0.92, 1.56)	0.93 (0.65, 1.31)
Exposure to intimate partner violence	16.4 (2663)	42.8 (15)	3.35** (1.41, 7.97)	2.95* (1.23, 7.03)	2.08 (0.94, 4.56)	19.3 (3975)	30.1 (71)	1.52 (0.97, 2.37)	1.37 (0.89, 2.11)	1.15 (0.67, 1.98)
Any child maltreatment	49.4 (7897)	70.2 (25)	2.13 (0.89, 5.09)	1.76 (0.73, 4.23)	—	48.3 (9788)	63.2 (152)	1.70** (1.19, 2.42)	1.45* (1.02, 2.04)	—

*Note*. All *n* values were unweighted, and all percentages were weighted. AOR‐1, adjusted for household income, age (continuous), marital status, race/ethnicity, education, and family history of dysfunction. AOR‐2, adjusted for household income, age (continuous), marital status, race/ethnicity, education, family history of dysfunction, and any mental disorder. AOR‐3, adjusted for household income, age (continuous), marital status, race/ethnicity, education, family history of dysfunction, any mental disorder, and all other types of child maltreatment.

*p ≤ .05; **p ≤ .01; ***p ≤ .001.

Among women, harsh physical punishment, physical abuse, sexual abuse, emotional abuse, physical neglect, and any child maltreatment were associated with increased odds of AN (AOR‐1 ranged from 1.35 to 2.20). When further adjusting for any mental disorder, odds ratios for physical abuse, sexual abuse, emotional abuse, and any child maltreatment remained significant (AOR‐2 ranged from 1.45 to 1.89). When also adjusting for the co‐occurrence of child maltreatment types, only sexual abuse remained significantly associated with AN among women (AOR‐3 = 1.77; 95% CI 1.13–2.79). Gender interaction effects were tested. The interaction terms for gender and sexual abuse (*p* = .016) and for gender and exposure to IPV (*p* = .019) were statistically significant indicating that the effects were stronger in men compared to women.

For BED (Table 4) among men, sexual abuse, physical neglect, and any child maltreatment were associated with increased odds of BED (AOR‐1 ranged from 2.21 to 2.26). In AOR‐2 models, the odds ratio for physical neglect remained statistically significant (AOR‐2 = 1.91; 95% CI 1.13–3.24) and remained significant in the AOR‐3 model (AOR‐3 = 1.88; 95% CI 1.08–3.29). Among women, all child maltreatment types were associated with increased odds of BED (AOR‐1 ranged from 1.83 to 3.29). In AOR‐2 models, odds ratios remained significant (AOR‐2 ranged from 1.57 to 2.57). In the AOR‐3 models further adjusting for all individual child maltreatment types, sexual abuse and emotional abuse remained independently associated with increased odds of BED (AOR‐3 = 1.60 and 1.64, respectively). Gender interaction effects were not statistically significant in the BED models.

**Table 4:** Associations between types of child maltreatment and lifetime binge‐eating disorder in men and women

Child maltreatment type	Men					Women				
	Lifetime prevalence no binge‐eating disorder % (*n*)	Lifetime prevalence binge‐eating disorder % (*n*)	AOR‐1	AOR‐2	AOR‐3	Lifetime prevalence no binge‐eating disorder % (*n*)	Lifetime prevalence binge‐eating disorder % (*n*)	AOR‐1	AOR‐2	AOR‐3
Harsh physical punishment	18.4 (2972)	28.4 (26)	1.37 (0.78, 2.41)	1.11 (0.63, 1.97)	0.97 (0.52, 1.84)	17.5 (3599)	38.7 (79)	2.29*** (1.62, 3.25)	1.83*** (1.29, 2.59)	0.88 (0.57, 1.38)
Physical abuse	18.0 (2902)	26.5 (24)	1.25 (0.72, 2.16)	1.00 (0.58, 1.71)	0.63 (0.34, 1.20)	16.9 (3492)	42.2 (84)	2.84*** (2.06, 3.93)	2.31*** (1.67, 3.19)	1.41 (0.87, 2.29)
Sexual abuse	6.0 (982)	14.7 (11)	2.21* (1.04, 4.73)	1.84 (0.87, 3.90)	1.76 (0.87, 3.58)	15.9 (3226)	38.6 (78)	2.57*** (1.77, 3.72)	2.02*** (1.40, 2.92)	1.60* (1.09, 2.33)
Emotional abuse	8.8 (1464)	19.7 (19)	1.81 (0.96, 3.38)	1.47 (0.79, 2.73)	1.33 (0.65, 2.74)	11.4 (2322)	36.4 (74)	3.29*** (2.22, 4.88)	2.57*** (1.74, 3.81)	1.64* (1.04, 2.59)
Emotional neglect	8.8 (1483)	10.6 (8)	0.99 (0.42, 2.35)	0.92 (0.39, 2.19)	0.77 (0.28, 2.14)	11.0 (2372)	21.9 (43)	1.83** (1.25, 2.68)	1.57* (1.08, 2.29)	0.83 (0.53, 1.30)
Physical neglect	30.0 (4816)	52.7 (46)	2.25** (1.32, 3.83)	1.91* (1.13, 3.24)	1.88* (1.08, 3.29)	25.4 (5219)	50.6 (105)	2.40*** (1.68, 3.42)	2.02*** (1.43, 2.86)	1.35 (0.91, 2.02)
Exposure to intimate partner violence	16.4 (2651)	30.0 (27)	1.49 (0.82, 2.71)	1.30 (0.72, 2.36)	1.22 (0.61, 2.42)	19.2 (3957)	43.2 (89)	2.30*** (1.58, 3.34)	1.94*** (1.35, 2.79)	1.38 (0.88, 2.15)
Any child maltreatment	49.3 (7861)	71.7 (61)	2.26* (1.21, 4.20)	1.78 (0.95, 3.36)	—	48.3 (9784)	73.4 (156)	2.35*** (1.53, 3.59)	1.82** (1.19, 2.79)	—

*Note*. All *n* values were unweighted, and all percentages were weighted. AOR‐1, adjusted for household income, age (continuous), marital status, race/ethnicity, education, and family history of dysfunction. AOR‐2, adjusted for household income, age (continuous), marital status, race/ethnicity, education, family history of dysfunction, and any mental disorder. AOR‐3, adjusted for household income, age (continuous), marital status, race/ethnicity, education, family history of dysfunction, any mental disorder, and all other types of child maltreatment.

*p ≤ .05; **p ≤ .01; ***p ≤ .001.

For BN (Table 5) among men, physical abuse, sexual abuse, emotional neglect, and exposure to IPV were associated with increased odds of BN (AOR‐1 ranged from 3.42 to 9.55). Among women, sexual abuse, emotional abuse, and emotional neglect were associated with increased odds of BN (AOR‐1 ranged from 2.21 to 2.52). In AOR‐2 models, odds ratios remained significant for emotional abuse and emotional neglect (AOR‐2 were 2.04 and 2.03, respectively). However, only emotional abuse remained significantly associated with BN in the fully adjusted models (AOR‐3 = 2.78; 95% CI 1.33–5.79). Gender interaction effects were not computed in the BN models because of inadequate statistical power.

**Table 5:** Associations between types of child maltreatment and lifetime bulimia nervosa in men and women

	Men					Women				
Child maltreatment type	Lifetime prevalence no bulimia nervosa % (*n*)	Lifetime prevalence bulimia nervosa % (*n*)	AOR‐1	AOR‐2	AOR‐3	Lifetime prevalence no bulimia nervosa % (*n*)	Lifetime prevalence bulimia nervosa % (*n*)	AOR‐1	AOR‐2	AOR‐3
Harsh physical punishment	18.5 (2993)	34.9 (5)	1.31 (0.31, 5.52)	—	—	17.6 (3651)	33.0 (27)	1.95 (0.996, 3.83)	1.60 (0.82, 3.13)	0.71 (0.28, 1.80)
Physical abuse	18.0 (2920)	54.3 (6)	3.42* (1.05, 11.15)	—	—	17.2 (3552)	26.7 (24)	1.39 (0.72, 2.65)	1.15 (0.60, 2.18)	0.93 (0.41, 2.12)
Sexual abuse	6.0 (989)	45.3 (4)	9.55** (2.22, 41.06)	—	—	16.1 (3276)	31.9 (28)	2.21* (1.17, 4.16)	1.78 (0.95, 3.34)	1.34 (0.66, 2.73)
Emotional abuse	8.8 (1479)	29.2 (4)	2.10 (0.41, 10.78)	—	—	11.6 (2368)	29.9 (28)	2.52** (1.32, 4.81)	2.04* (1.08, 3.83)	2.78** (1.33, 5.79)
Emotional neglect	8.8 (1486)	52.1 (5)	8.58** (1.81, 40.60)	—	—	11.0 (2391)	24.1 (24)	2.32* (1.19, 4.49)	2.03* (1.05, 3.95)	1.72 (0.80, 3.68)
Physical neglect	30.1 (4857)	51.2 (5)	1.85 (0.58, 5.92)	—	—	25.6 (5290)	41.2 (34)	1.61 (0.85, 3.05)	1.37 (0.72, 2.60)	1.26 (0.57, 2.76)
Exposure to intimate partner violence	16.4 (2671)	62.1 (7)	4.91* (1.37, 17.64)	—	—	19.4 (4022)	32.5 (24)	1.42 (0.70, 2.91)	1.25 (0.62, 2.52)	0.64 (0.34, 1.21)
Any child maltreatment	49.4 (7914)	68.9 (8)	1.37 (0.32, 5.92)	—	—	48.5 (9887)	68.2 (53)	1.88 (0.99, 3.57)	1.50 (0.79, 2.86)	—

*Note*. All *n* values were unweighted, and all percentages were weighted. AOR‐1, adjusted for household income, age (continuous), marital status, race/ethnicity, education, and family history of dysfunction. AOR‐2, adjusted for household income, age (continuous), marital status, race/ethnicity, education, family history of dysfunction, and any mental disorder. AOR‐3, adjusted for household income, age (continuous), marital status, race/ethnicity, education, family history of dysfunction, any mental disorder, and all other types of child maltreatment.

*p ≤ .05; **p ≤ .01; ***p ≤ .001.

— Indicates that models were not computed because of inadequate power and lack of variance.

Finally, among respondents with only one lifetime eating disorder, harsh physical punishment, physical abuse, sexual abuse, emotional abuse, and exposure to IPV were all equally associated with each individual eating disorder (with a few exceptions for BN likely because of underpowered models) (Table 6). Emotional neglect was more strongly associated with BN and BED than with AN. Physical neglect, any child maltreatment, and a family history of dysfunction were more strongly associated with BED compared to AN, but not statistically different from BN.

**Table 6:** Multinomial regression between types of child maltreatment and types of eating disorders in the total sample

		Type of lifetime eating disorder			
Child maltreatment type	Estimate	No eating disorder	Anorexia nervosa	Bulimia nervosa	Binge eating disorder
Harsh physical punishment	% (*n*)	17.8 (6468)	25.8 (72)	30.6 (24)	35.1 (100)
	AOR (95% CI)	1.00	1.64^a^ (1.17, 2.30)**	2.08^a^ (1.04, 4.18)*	2.52^a^ (1.86, 3.40)***
Physical abuse	% (*n*)	17.3 (6290)	28.0 (75)	23.0 (21)	37.0 (103)
	AOR (95% CI)	1.00	1.91^a^ (1.33, 2.74)***	1.46^a^ (0.79, 2.72)	2.83^a^ (2.12, 3.79)***
Sexual abuse	% (*n*)	11.0 (4113)	26.3 (64)	28.2 (22)	29.7 (83)
	AOR (95% CI)	1.00	2.11^a^ (1.46, 3.04)***	2.43^a^ (1.23, 4.80)*	3.05^a^ (2.20, 4.24)***
Emotional abuse	% (*n*)	10.0 (3696)	21.6 (59)	26.5 (24)	29.8 (87)
	AOR (95% CI)	1.00	2.25^a^ (1.53, 3.33)***	2.97^a^ (1.59, 5.56)***	3.67^a^ (2.61, 5.15)***
Emotional neglect	% (*n*)	9.9 (3788)	12.3 (38)	27.4 (23)	18.4 (49)
	AOR (95% CI)	1.00	1.18^a^ (0.81, 1.71)	3.18^b^ (1.66, 6.09)***	1.99^b^ (1.43, 2.76)***
Physical neglect	% (*n*)	27.5 (9904)	35.6 (93)	38.5 (28)	51.5 (146)
	AOR (95% CI)	1.00	1.60^a^ (1.21, 2.11)***	1.79^a,b^ (0.95, 3.40)	2.92^b^ (2.20, 3.87)***
Exposure to intimate partner violence	% (*n*)	17.7 (6505)	28.9 (72)	32.1 (22)	38.6 (111)
	AOR (95% CI)	1.00	1.76^a^ (1.23, 2.53)**	2.06^a^ (1.01, 4.20)*	2.84^a^ (2.13, 3.79)***
Any child maltreatment	% (*n*)	48.6 (17428)	62.4 (157)	65.6 (46)	73.6 (210)
	AOR (95% CI)	1.00	1.78^a^ (1.28, 2.48)***	2.05^a,b^ (1.001, 4.19)*	2.98^b^ (2.20, 4.03)***
Any family dysfunction	% (*n*)	29.0 (10428)	42.8 (107)	54.4 (35)	55.6 (151)
	AOR (95% CI)	1.00	1.72^a^ (1.29, 2.28)***	2.75^a,b^ (1.50, 5.04)***	2.98^b^ (2.16, 4.11)***

*Note*. All *n* values were unweighted, and all percentages were weighted. Individuals with more than one eating disorder were removed from analyses. AOR = adjusted for sex. Estimates with different superscripts are significantly different from one another at *p* ≤ .05.

*p ≤ .05; **p ≤ .01; ***p ≤ .001.

## 4 DISCUSSION

The current study advances knowledge on child maltreatment and eating disorders in a number of novel ways. First, all child maltreatment types were all associated with eating disorders with important gender difference noted in these relationships. Among men, sexual abuse and physical neglect had the most robust relationship with eating disorders. Among women, sexual abuse and emotional abuse had the most robust relationships with eating disorders. Second, physical neglect and any family history of dysfunction were more strongly associated with BED compared to AN. As well, emotional neglect was more strongly associated with BN and BED compared to AN. In addition to these novel findings, the data also confirm two previous findings in the literature. First, the prevalence of lifetime eating disorders are higher among women compared to men (Hudson et al., 2007). Second, with few significant findings for sociodemographic characteristics, the data indicate that eating disorders are not more likely among certain sociodemographic profiles, despite common stereotypes (Becker, Hadley Arrindell, Perloe, Fay, & Striegel‐Moore, 2010). This is consistent with previous findings (Hudson et al., 2007; Kessler et al., 2013).

Previous population‐based studies examining child maltreatment and eating disorders have found that sexual abuse and physical abuse are related to an increased likelihood of experiencing eating disorders or symptoms (Garfinkel et al., 1995; Micali et al., 2017; Sachs‐Ericsson et al., 2012). Our findings are consistent with the current literature, but also extend knowledge. In our study, all seven child maltreatment types were associated with individual eating disorders and having any eating disorder with few exceptions noted for BN, which was likely because of underpowered models. Importantly, notable gender differences were found. Sexual abuse and physical neglect had the most robust relationship with any eating disorder among men, while sexual abuse and emotional abuse were the most robust among women. With regard to AN, significant relationships were noted for many child maltreatment types. However, sexual abuse had the most robust relationship with AN, and this effect was statistically stronger among men compared to women. For BED, a different trend was noted among men and women. Among women, all seven child maltreatment types were associated with increased odds of BED compared to only two child maltreatment types among men (sexual abuse and physical neglect). Different trends were also noted for BED in the most adjusted models with physical neglect having the most robust relationship among men and sexual abuse and emotional abuse having the most robust relationships among women. These are important novel findings since other representative community studies have not examined gender differences (Garfinkel et al., 1995; Micali et al., 2017; Sachs‐Ericsson et al., 2012). As well, the data indicate that some child maltreatment types are more strongly associated with certain eating disorders with emotional neglect being more strongly associated with BN and BED compared to AN, and physical neglect being more strongly associated with BED compared to AN. These findings may have clinical relevance for understanding potential risk for specific eating disorders following child maltreatment.

Notably, when comparing the prevalence of eating disorders in the current study to previous epidemiologic data, some similarities and difference were found. The lifetime prevalence of AN is similar (0.6% in national comorbidity survey‐replication [NCS‐R] and 0.8% in the NESARC‐III), while lifetime prevalence of BN (0.2% and 1.0%) and BED (0.8% and 2.8%) were lower in the current study compared to previous data (Hudson et al., 2007). These differences warrant consideration since the NCS‐R data used the stricter DSM‐IV criteria with regard to number of binge‐eating episodes (i.e., a binge‐eating episode at least twice a week for 3 months), while the NESARC‐III used DSM‐5 criteria (i.e., a binge‐eating episode at least once a week for 3 months). Two possible reasons may account for the lower prevalence of BN and BED in the current study. First, the criteria for binge‐eating episode in the NESARC‐III data specifically excluded eating an unusually large amount of food that occurred during the holidays. This exclusion was not provided in the NCS‐R survey. Second, the NCS‐R data assessed more indicators of distress for BED. Because of these differences, we believe that the current DSM‐5 eating disorders may be more conservative estimates of eating disorder prevalence, but remain reliable. Importantly, however, it must be stated that the NCS‐R and the NESARC were collected using different methods in different years. It may not actually be possible to make direct comparisons of these data because of these differences.

It is important to also recognize limitations of the current study. First, the data were cross‐sectional, which does not allow for the determination of causal inferences. Second, child maltreatment and family history of dysfunction data were collected retrospectively, which could introduce error because of reporting and recall bias. However, there is evidence that indicates valid recall of adverse childhood events (Hardt & Rutter, 2004) and that psychopathology is not related to less reliable reporting of such events (Brewin, Andrews, & Gotlib, 1993). As well, family history of dysfunction did not include all adverse events that may have been important to include. Third, data on exposure to IPV were only assessed for violence against women. This is a limitation since both men and women experience IPV (Afifi et al., 2009). Fourth, data were collected by lay interviewers rather than clinicians. Fifth, even though our sample was large, some models were still underpowered resulting in the inability to run all BN models among men. Finally, although the reliability and validity of AUDADIS‐5 for assessing DSM‐5 diagnoses for substance use, mood, anxiety, personality, and trauma and stress‐related disorders have been found to be reliable and valid (Grant, Goldstein, Smith, et al., 2015; Hasin, Greenstein, et al., 2015; Hasin, Shmulewitz, et al., 2015), specific data for eating disorders were not included because of the low prevalence of eating disorders and inability to run the statistical tests. However, we have no reason to believe that the reliability and validity of the AUDADIS‐5 would be any different for eating disorders.

It is known from clinical data that patients with an eating disorder report higher prevalence of child maltreatment (Becker & Grilo, 2011; Smolak & Murnen, 2002). The current study adds to this with the important finding from general population data that all child maltreatment types, including harsh physical punishment, physical abuse, sexual abuse, emotional abuse, emotional neglect, physical neglect, and exposure to IPV, are associated with increased odds of eating disorders among men and women. It is also important for clinicians to be aware that these relationships may vary according to gender, since previous studies have not adequately examined gender differences with regard to child maltreatment experiences. The robust relationship in the most adjusted models indicated that sexual abuse is strongly associated with increased odds of eating disorders among men and women. This is consistent with past research (Chen et al., 2010). However, robust relationships also existed for physical neglect and eating disorders among men and emotional abuse and eating disorders among women.

Furthermore, although much of the previous research to date has been conducted on physical or sexual abuse, it is important to recognize that experiences of harsh physical punishment, emotional abuse, emotional neglect, physical neglect, and exposure to IPV are all associated with increased odds of eating disorders. From a policy perspective, it is important to acknowledge the large and robust relationships between child maltreatment and eating disorders in the general population. It is possible that effective strategies to reduce child maltreatment may also correspond with reductions in eating disorders. Also, it is necessary for public policies to be inclusive of men, while being aware of the gender differences according to child maltreatment type and specific eating disorders.

## CONFLICT OF INTEREST

The authors indicate no conflict of interest.
